# Identification of potential key genes and pathways predicting pathogenesis and prognosis for triple-negative breast cancer

**DOI:** 10.1186/s12935-019-0884-0

**Published:** 2019-06-28

**Authors:** Xuemei Lv, Miao He, Yanyun Zhao, Liwen Zhang, Wenjing Zhu, Longyang Jiang, Yuanyuan Yan, Yue Fan, Hongliang Zhao, Shuqi Zhou, Heyao Ma, Yezhi Sun, Xiang Li, Hong Xu, Minjie Wei

**Affiliations:** 1 Department of Pharmacology, School of Pharmacy, China Medical University, No. 77 Puhe Road, Shenyang North New Area, Shenyang, 110122 Liaoning Province People’s Republic of China; 2 Liaoning Key Laboratory of Molecular Targeted Anti-tumour Drug Development and Evaluation, Shenyang, China; 3 Department of Breast Cancer, Cancer Hospital of China Medical University, No. 44, Xiaoheyan Road, Dadong District, Shenyang, 110042 China

**Keywords:** Triple-negative breast cancer, Differentially expressed genes, Prognostic, Survival, mRNA-signature, Pathogenesis

## Abstract

**Background:**

Triple negative breast cancer (TNBC) is a specific subtype of breast cancer with a poor prognosis due to its aggressive biological behaviour and lack of therapeutic targets. We aimed to explore some novel genes and pathways related to TNBC prognosis through bioinformatics methods as well as potential initiation and progression mechanisms.

**Methods:**

Breast cancer mRNA data were obtained from The Cancer Genome Atlas database (TCGA). Differential expression analysis of cancer and adjacent cancer, as well as, triple negative breast cancer and non-triple negative breast cancer were performed using R software. The key genes related to the pathogenesis were identified by functional and pathway enrichment analysis and protein–protein interaction network analysis. Based on univariate and multivariate Cox proportional hazards model analyses, a gene signature was established to predict overall survival. Receiver operating characteristic curve was used to evaluate the prognostic performance of our model.

**Results:**

Based on mRNA expression profiling of breast cancer patients from the TCGA database, 755 differentially expressed overlapping mRNAs were detected between TNBC/non-TNBC samples and normal tissue. We found eight hub genes associated with the cell cycle pathway highly expressed in TNBC. Additionally, a novel six-gene (*TMEM252*, *PRB2*, *SMCO1*, *IVL*, *SMR3B* and *COL9A3*) signature from the 755 differentially expressed mRNAs was constructed and significantly associated with prognosis as an independent prognostic signature. TNBC patients with high-risk scores based on the expression of the 6-mRNAs had significantly shorter survival times compared to patients with low-risk scores (*P *< 0.0001).

**Conclusions:**

The eight hub genes we identified might be tightly correlated with TNBC pathogenesis. The 6-mRNA signature established might act as an independent biomarker with a potentially good performance in predicting overall survival.

**Electronic supplementary material:**

The online version of this article (10.1186/s12935-019-0884-0) contains supplementary material, which is available to authorized users.

## Background

Triple-negative breast cancer (TNBC) is defined as a subtype of aggressive breast cancer, accounting for 10–20% of all breast cancer cases [1]. TNBC subjects lack expression of the estrogen receptor (ER) and progesterone receptor (PR) and does not amplify the human epidermal growth factor receptor 2 (HER2) [2]. TNBC is more commonly diagnosed among young women and is more prone to relapse and visceral metastasis, compared with other breast cancer subtypes [3–5**]**. Due to the absence of molecular targets, patients diagnosed with TNBC cannot receive endocrine or HER2 targeted therapy [6], increasing the difficulty of treatment for them [7]. Chemotherapy is still the main adjuvant treatment option for patients with TNBC [8]. TNBC remains a disease associated with poor prognosis and limited treatment options because many tumours are resistant to chemotherapy and rapidly relapse or metastasize after adjuvant therapy [9]. The identification of uniform targets can help achieve more effective and less toxic treatment. Hence, it is imperative and urgent to explore new therapeutic targets for TNBC [10].

Recently, many biomarkers have been developed for breast cancer. For example, CD82, a potential diagnostic biomarker for breast cancer [11]. Furthermore, seven lncRNAs (MAGI2-AS3, GGTA1P, NAP1L2, CRABP2, SYNPO2, MKI67, and COL4A6) detected to be associated with TNBC prognosis, can be promising biomarkers [12]. Advancements in microarray and high throughput sequencing technologies have provided efficient tools to help in developing more reliable biomarkers for diagnosis, survival and prognosis [13, 14]. However, the predictive power of a single gene biomarker may be insufficient. Emerging studies have found that gene signatures, including several genes, may be better alternatives [15]. To the best of our knowledge, the studies about multi-gene prognostic signatures in TNBC are very few, and the functions and mechanisms of mRNAs in TNBC remain to be further explored. Thus, it is necessary to identify more sensitive and efficient mRNA signatures for TNBC prognosis.

In this study, we first identified differentially expressed genes (DEGs), using 1109 BC samples and 113 matched non-cancerous samples from The Cancer Genome Atlas (TCGA). We identified ten hub genes associated with the cell cycle by functional enrichment analysis, protein–protein interaction (PPI) network and survival analysis. In addition, we developed a novel six-gene signature that could effectively predict TNBC survival.

## Methods

### Collection of clinical specimen data from the TCGA and GEO databases

The mRNA expression profiles and corresponding clinical information of breast cancer patients were downloaded from the Cancer Genome Atlas (TCGA) and gene expression omnibus (GEO) databases. We collected 1109 samples with gene expression data, containing 1109 BC tumour tissues samples and 113 normal tissue samples from TCGA database. After removing patients with incomplete information, we were left with117 TNBC samples and 970 non-TNBC samples. We collected 270 samples with 58 normal breast tissue samples and 212 TNBC tissue samples from the GEO dataset of the NCBI GEO database (GSE31519, GSE9574, GSE20194, GSE20271, GSE45255, and GSE15852).

### Identification of differentially expressed genes

First, we merged the RNA-sequencing (RNA-seq) dataset files into a matrix file using the Perl language merge script. The gene name was converted from an Ensembl id to a gene symbol via the Ensembl database. Finally, the “edgeR” and “heatmap”R package were used to screen for differential genes between 117 TNBC and 970 other breast cancer patient subtypes and to map volcanoes. | log FC | > 1.0 and *P *< 0.05 were considered as the threshold value.

### Functional and pathway enrichment analysis

Gene Ontology (GO) analysis and Kyoto Encyclopedia of Genes and Genomes (KEGG) pathway enrichment analysis of DEGs were performed using Database for Annotation, Visualization and Integration Discovery, DAVID version 6.8 [16]. *P *< 0.05 was chosen as the cut-off criterion. GO is a set of unified vocabulary to describe molecular functions (MF), biological processes (BP) and cellular components (CC) of biology, whereas KEGG analysis was performed to aid understanding of the signalling pathways involving DEGs.

### PPI network construction and modules selection

A PPI network of differential genes was constructed, using STRING version 10.5 to evaluate information on protein–protein interactions [17]. Using the Molecular Complex Detection (MCODE) plug-in in Cytoscape 3.7.0, a visualization tool for integrating many molecular states such as expression level and interaction information into a unified conceptual framework [18], the PPI network module with densely connected regions was obtained (Degree cut-off > 15) [19].

### Survival analysis

Clinical characteristic information for breast cancer was downloaded from TCGA. After removing samples with incomplete clinical overlapping DEG data, samples from 117 TNBC patients were used for further analysis. Univariate and multivariate Cox model analyses were used to identify candidate genes that were significantly associated with overall survival (OS). Based on the expression level and coefficient (*β*) of each gene, calculated by multivariate Cox proportional hazards regression analysis, a novel reliable prognostic gene signature was established. These TNBC patient samples were further divided into low- or high-risk groups based on the median risk score as the cut-off point. Kaplan–Meier curves were used to assess the prognostic value of the risk score. In addition, a time-dependent receiver operating characteristic (ROC) curve analysis, using the R package “survivalROC” was constructed to assess the predictive accuracy of the gene signature for time-dependent cancer death [20]. The area under the curve (AUC) was calculated to evaluate the predictive ability of the gene signature for clinical outcomes.

## Results

### Identification of differentially expressed genes in TNBC

**Fig. 1:**
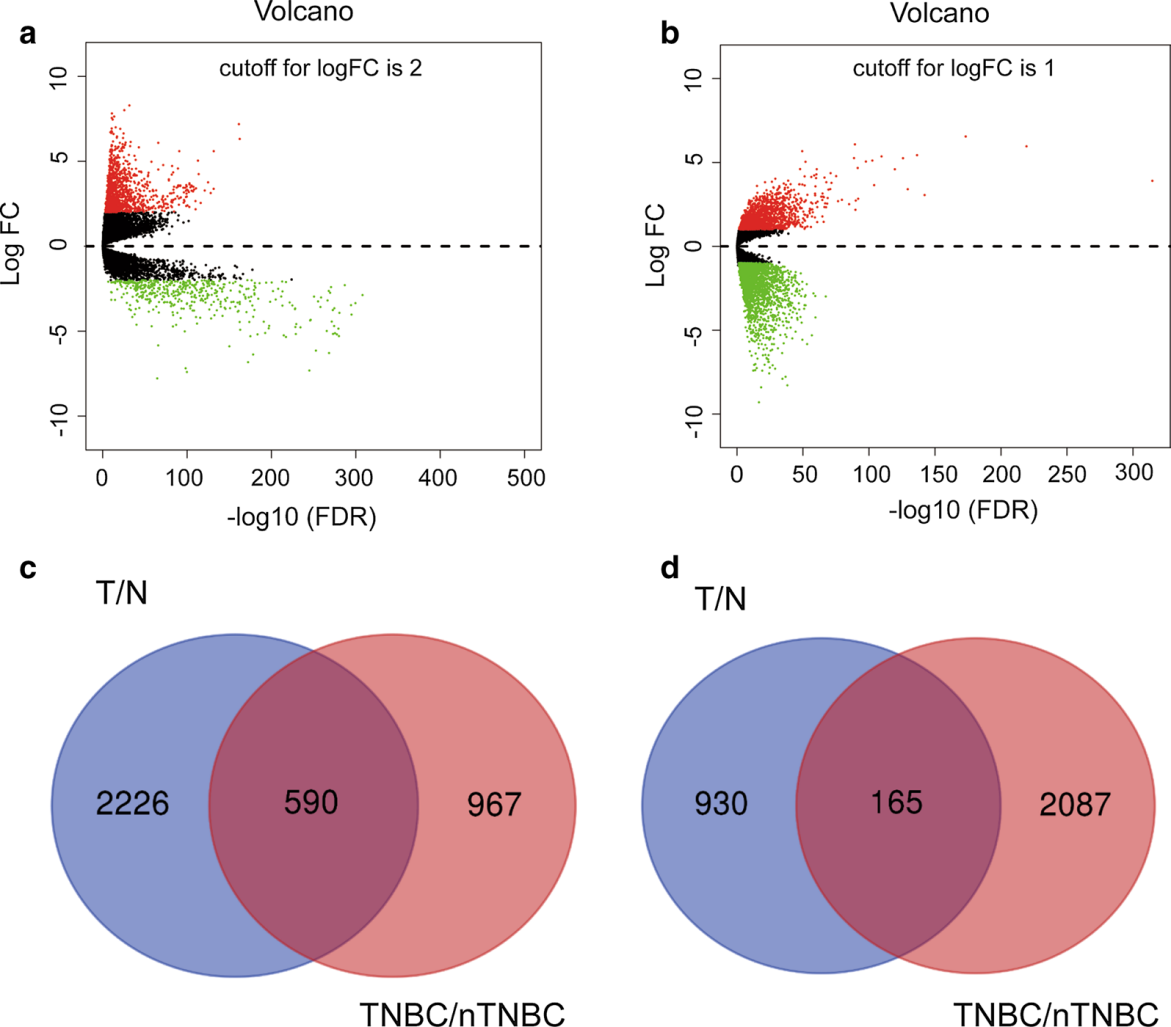
Identification of differentially expressed genes (DEGs) and Venn diagram of DEGs in triple-negative breast cancer (TNBC). Volcano plot of all genes **a** between 1109 breast cancer tissue samples and 113 normal tissue samples, and **b** between 117 TNBC and 970 non-TNBC breast cancer samples from TCGA database. Red dots represent upregulated genes, and green dots represent downregulated genes. **c** Venn diagram for overlapping upregulated genes and downregulated genes in the two sets. T: Tumour; N: normal

We used the “EDGR” and “Volcano” packages in the R software to identify differentially expressed genes between 1109 breast cancer tissue samples and 113 normal tissue samples from TCGA database (|logFC| ≥ 2 and adjusted *P *< 0.05), and screened out2816 up-regulated and 1095 down-regulated genes (Fig. 1a). We further analysed the DEGs between 117 TNBC and 970 non-TNBC breast cancer samples (|logFC| > 1 and adjusted *P *< 0.05), and identified a total of 1557 up-regulated genes and 2972 down-regulated genes (Fig. 1b). In addition, we used the Venn diagram web-tool (http://bioinformatics.psb.ugent.be/webtools/Venn/) to cross the two sets of differential genes and found 755 overlapped DEGs (Additional file 1: Table S1), including 590 up-regulated genes (Fig. 1c) and 165 down-regulated genes (Fig. 1d).

### GO term and KEGG pathway enrichment analysis of DEGs

**Fig. 2:**
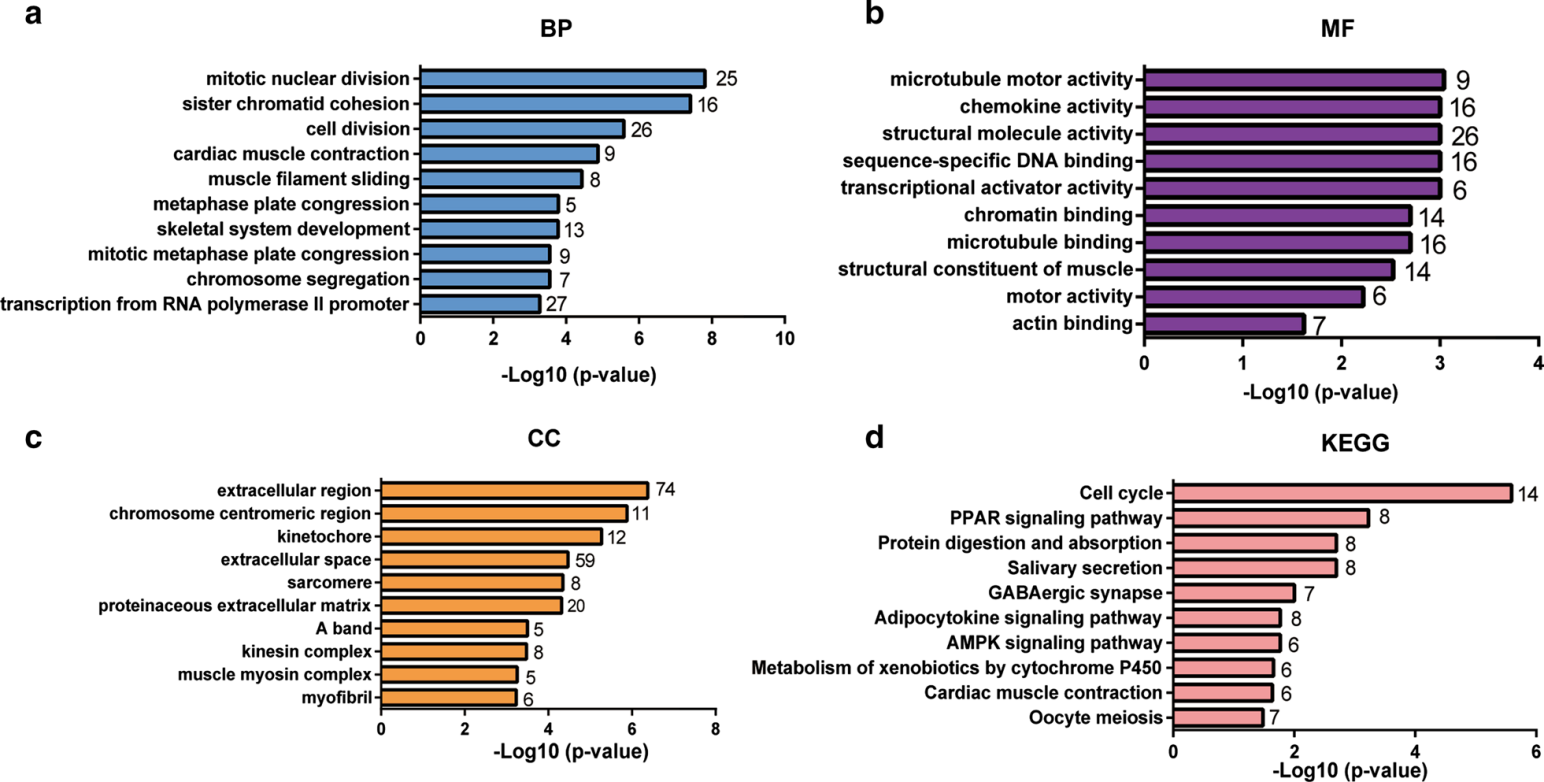
Top 10 functional enrichment analyses of the overlapping DEGs. **a** GO: Gene ontology; BP: biological process. **b** MF: molecular function. **c** CC: cellular component. **d** KEGG: Kyoto Encyclopedia of Genes and Genomes

GO function and KEGG pathway enrichment analysis were performed using DAVID to expound the biological functions of 755 DEGs (Additional file 2: Table S2). The BP results indicated that DEGs were mainly significantly enriched in mitotic nuclear division, sister chromatid cohesion, cell division (Fig. 2a). MF analysis showed that DEGs were significantly enriched in microtubule motor, chemokine and structural molecule activities (Fig. 2b). CC analysis showed that the DEGs were mainly enriched in the extracellular region, chromosome centromeric region and kinetochore (Fig. 2c). In addition, the most enriched KEGG pathways were PPAR signalling, AMPK signalling and oocyte meiosis pathways (Fig. 2d).

### A cell cycle related module selection by PPI Network analysis

**Fig. 3:**
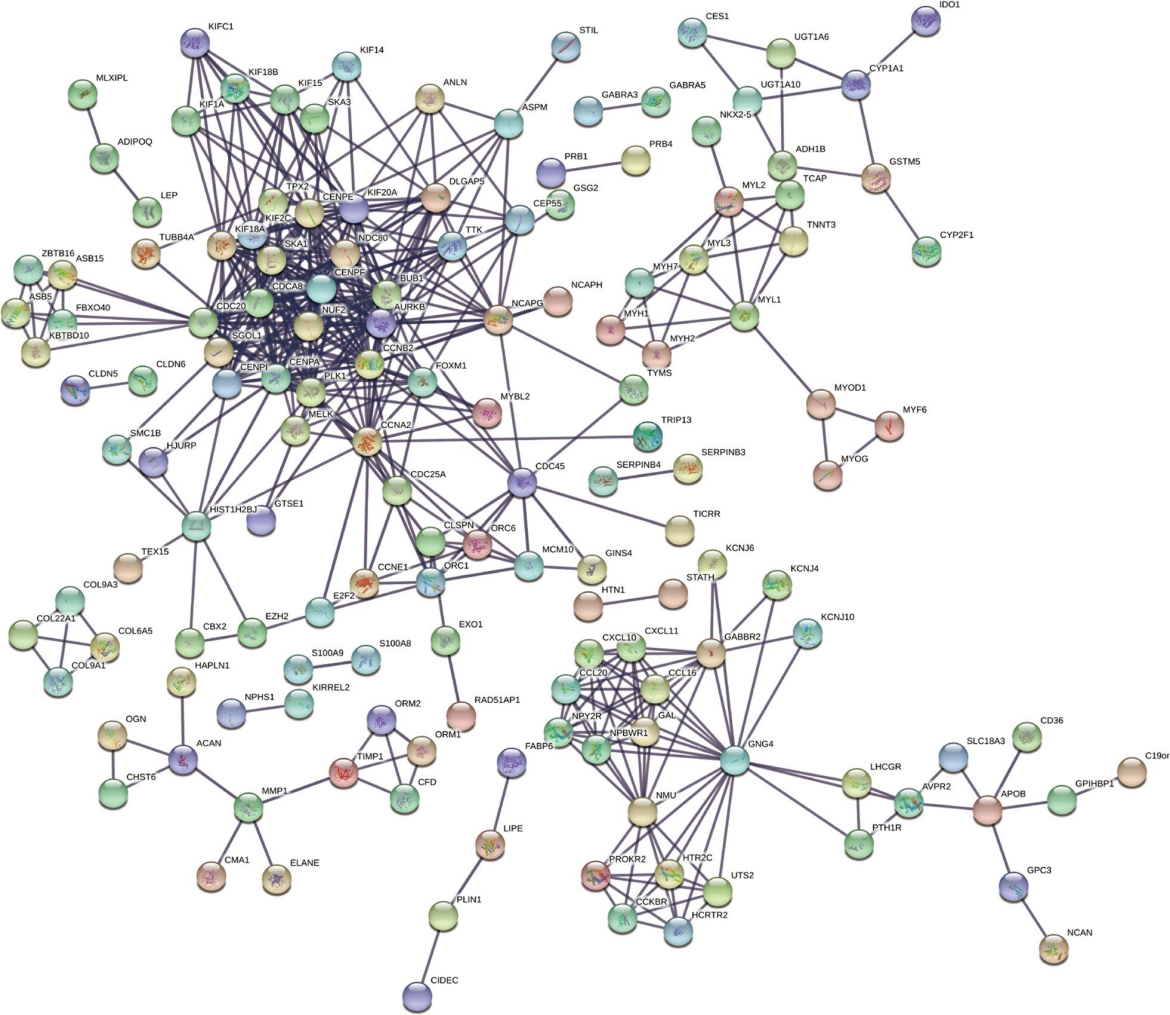
PPI network of DEGs. DEGs, differentially expressed genes; PPI: protein–protein interaction

**Fig. 4:**
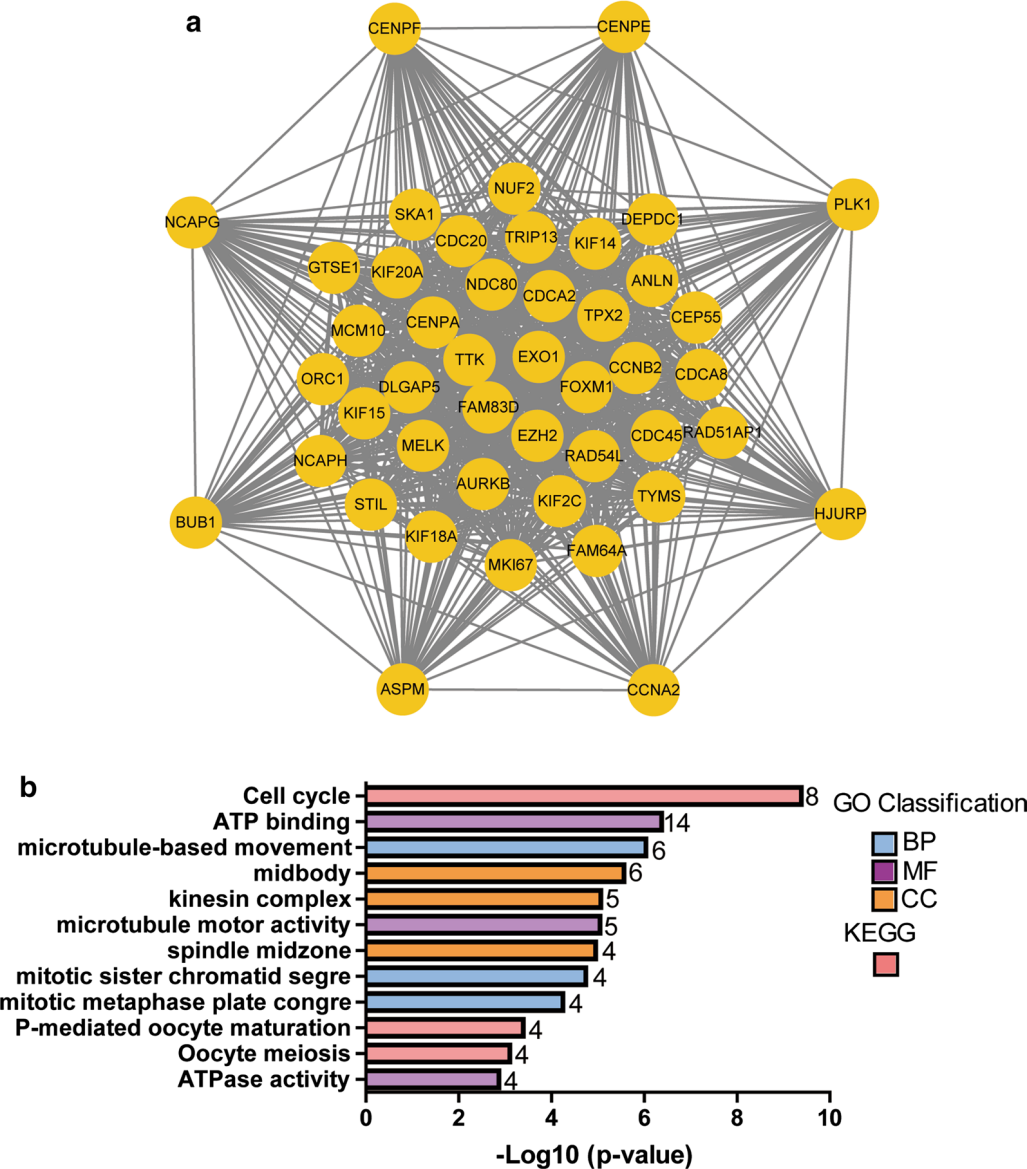
The module identified in the PPI network of the DEGs. **a** A significant module selected from the PPI network. **b** Functional and pathway enrichment analysis of the DEGs in the module

**Fig. 5:**
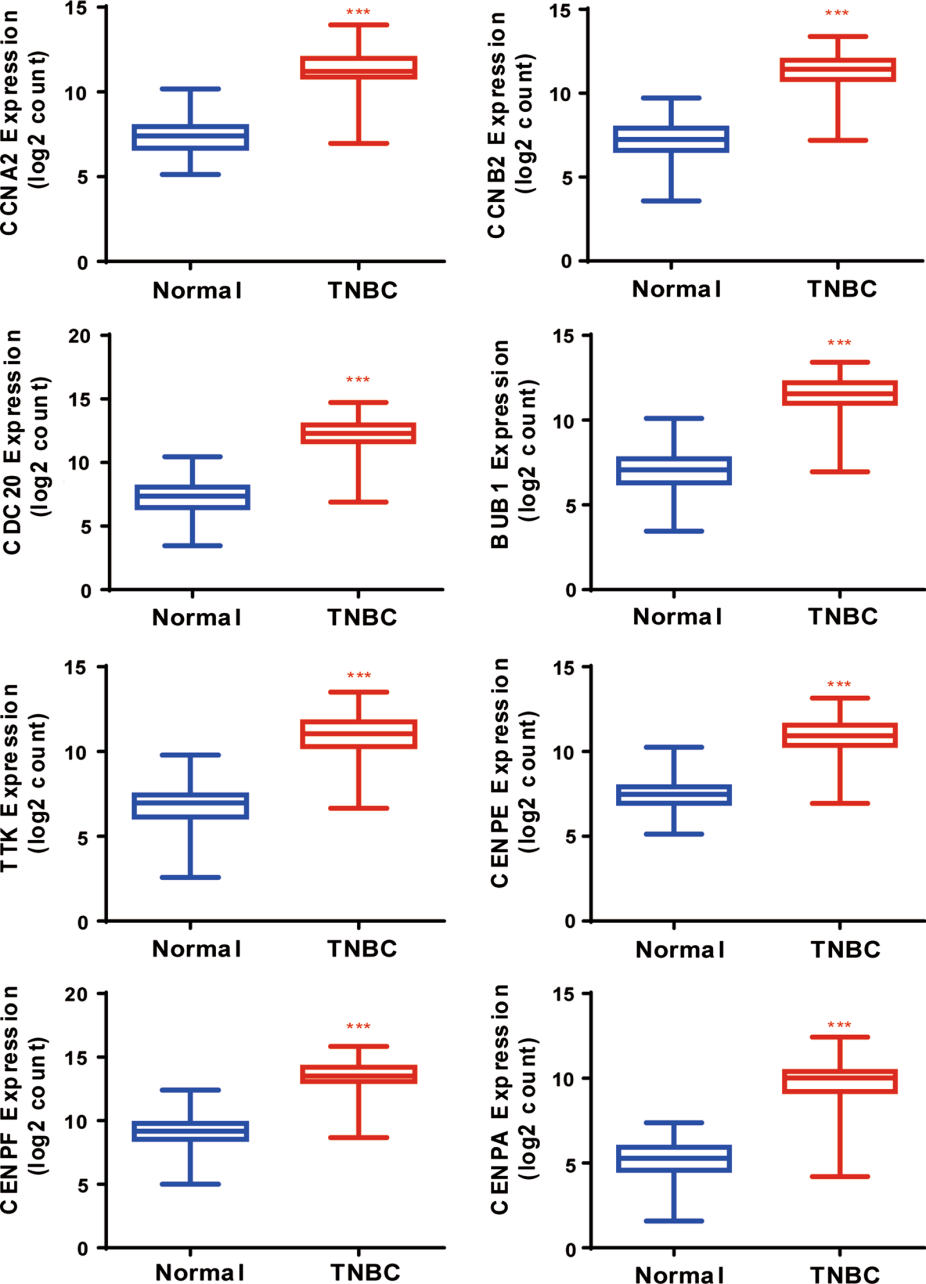
Expression of the eight hub genes correlated with the cell cycle in TNBC (TCGA dataset). Expression values of genes are log2-transformed

Protein interactions among overlapping DEGs were predicted with STRING tools. A total of 148 nodes and 477 edges were displayed in the PPI network (Fig. 3) with PPI-enrichment *P* value < 1.0e−16. A PPI network for DEG subsets with a combined score > 0.9 was constructed to determine the candidate hub genes. Based on the PPI network of the subsets, a module with an MCODE score of 42 and 45 nodes was identified (Fig. 4a), and functional enrichment analyses showed that the genes in this module were mainly associated with the cell cycle and mitosis (Fig. 4b and Table 1). BP analysis showed that these genes were significantly enriched in microtubule-based movement, mitotic sister chromatid segregation, mitotic metaphase plate congression, cell division, and mitotic cytokinesis. For CC analysis, these genes were significantly enriched in the condensed nuclear chromosome outer kinetochore, kinetochore, and spindle midzone. MF analysis showed the genes were significantly enriched in ATP binding, microtubule motor activity, single-stranded DNA binding, and DNA replication origin binding. In addition, the results of KEGG pathway enrichment analysis suggested that the pathways were enriched as follows: cell cycle, progesterone-mediated oocyte maturation, and oocyte meiosis. As a result, the eight genes correlated with cell cycle were selected as hub genes, which were CCNA2, CCNB2, CDC20, BUB1, TTK, CENPF, CENPA and CENPE (Table 2). Their expression levels were validated in 117 TNBC samples and 113 normal controls with breast cancer mRNA data from TCGA. As shown in Fig. 5, the eight mRNAs were significantly increased in TNBC compared with 113 normal control tissues (*P *< 0.001). We validated on the GEO database that the eight mRNA were also significantly increased compared with normal control tissues in TNBC (*P *< 0.001) (Additional file 3: Fig. S1).

**Table 1:** Functional and pathway enrichment analysis of the DEGs in module

Category	Term	Description	Count	*P*-value
BP term	GO:0007018	Microtubule-based movement	6	9.15E−07
BP term	GO:0000070	Mitotic sister chromatid segregation	4	1.81E−05
BP term	GO:0007080	Mitotic metaphase plate congression	4	5.69E−05
BP term	GO:0051301	Cell division	4	3.19E−04
BP term	GO:0000281	Mitotic cytokinesis	3	0.001
CC term	GO:0030496	Midbody	6	2.73E−06
CC term	GO:0005871	Kinesin complex	5	8.61E−06
CC term	GO:0051233	Spindle midzone	4	1.11E−05
CC term	GO:0000942	Condensed nuclear chromosome outer kinetochore	3	2.29E−05
CC term	GO:0000776	Kinetochore	4	4.57E−04
MF term	GO:0005524	ATP binding	14	4.19E−07
MF term	GO:0003777	Microtubule motor activity	5	9.01E−06
MF term	GO:0016887	ATPase activity	4	0.001
MF term	GO:0003697	Single-stranded DNA binding	3	0.006
MF term	GO:0003688	DNA replication origin binding	2	0.020
KEGG pathway	cfa04110	Cell cycle	8	4.13E−10
KEGG pathway	cfa04914	Progesterone-mediated oocyte maturation	4	4.04E−04
KEGG pathway	cfa04114	Oocyte meiosis	4	7.89E−04

BP: Biological process; CC: cellular component; DEGs: differentially expressed genes; GO: Gene Ontology; KEGG: Kyoto Encyclopedia of Genes and Genomes; MF: molecular function

**Table 2:** The eight hub genes correlated with the cell cycle

Gene	Full name	Degree	Regulation
CCNA2	CyclinA2	26	UP
CCNB2	CyclinB2	23	UP
CDC20	Cell division cycle 20	25	UP
BUB1	BUB1 mitotic checkpoint serine	27	UP
TTK	TTK protein kinase	15	UP
CENPE	Centromere protein E	26	UP
CENPF	Centromere protein F	20	UP
CENPA	Centromere protein A	21	UP

Using Cox proportional hazards regression model, we analysed the genes in the module, but no significant gene signature was established to predict overall survival.

### Construction of a six-mRNA signature for survival prediction

**Fig. 6:**
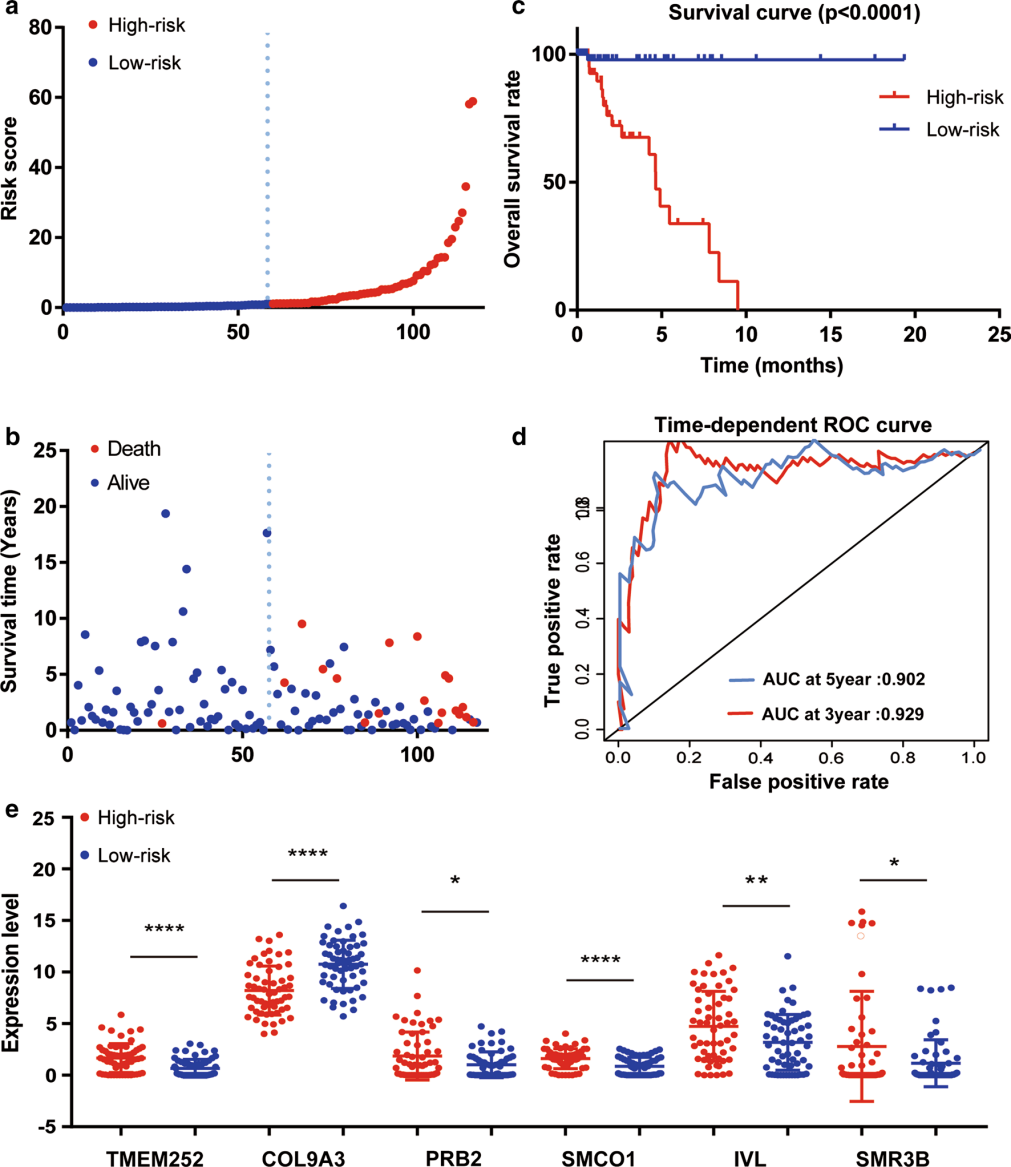
Prognostic gene signature of the six genes in 117 TNBC patients. **a** Risk score distribution; **b** patients’ survival status distribution; **c** Kaplan–Meier curves for low-risk and high-risk groups; **d** time-dependent ROC curves for predicting OS in TNBC patients by the risk score; **e** expression of the six genes in low- and high-risk groups (TCGA dataset). Gene expression values are log2-transformed

A total of 16 out of 755 DEGs were significantly correlated with survival time (*P* < 0.05) and identified by the univariate Cox proportional hazards regression model (Additional file 2: Table S3). Additionally, a prognostic gene signature, composed of six genes, was developed after using the multivariate Cox proportional hazards regression model. The genes include transmembrane protein 252 (TMEM252), collagen type IX alpha 3 chain (COL9A3), proline rich protein BstNI subfamily 2 (PRB2), single-pass membrane protein with coiled-coil domains 1 (SMCO1), involucrin (IVL), and submaxillary gland androgen regulated protein 3B (SMR3B) (Table 3). Patients were divided into low- and high-risk groups by the median risk score (1.070) (risk score = expression of SMR3B × 1.2141 + expression of TMEM252 × 1.6187 + expression of PRB2 × 1.4416 + expression of PRB2 × 2.0147 + expression of SMCO1 × 1.1471 + expression of COL9A3 × − 0.6101). The six-gene-based risk score distribution was presented in Fig. 6a. A highly significant difference in overall survival (OS) was detected between high- and low-risk groups (*P* < 0.0001) as shown in Fig. 6b. Moreover, the survival rate of the high-risk group was significantly much lower than for the low-risk group as depicted by Kaplan–Meier analysis in Fig. 6c (*P* < 0.0001). Time dependent ROC curve revealed that the prognostic signature presented a good performance in survival prediction, as shown in Fig. 6d and that the AUC was 0.929for 3 years OS and 0.902 for 5 years. Expression levels of the six genes in low- and high-risk groups are shown in Fig. 6e.

**Table 3:** Prognostic values for the six genes in 117 TNBC patients that make up the prognostic gene signature

Gene symbol	Univariate analysis		Multivariate analysis		
	HR (95% CI)	*P*-value	HR (95% CI)	*P*-value	Coefficient
SMR3B	1.125 (1.031–1.228)	0.0080	1.2141 (1.085–1.359)	0.0007	0.1940
TMEM252	1.452 (1.047–2.014)	0.0255	1.6187 (1.052–2.492)	0.0286	0.4816
PRB2	1.263 (1.020–1.564)	0.0318	1.4416 (1.117–1.860)	0.0049	0.3657
SMCO1	1.848 (1.080–3.162)	0.0249	2.0147 (1.176–3.451)	0.0107	0.7005
IVL	1.135 (1.007–1.281)	0.0387	1.1471 (0.980–1.343)	0.0878	0.1373
COL9A3	0.789 (0.649–0.960)	0.0178	0.6101 (0.464–0.802)	0.0003	− 0.4942

HR: Hazard ratio; CI: confidence interval

### 6-mRNA signature act as an independent prognostic indicator

Using univariate and multivariate Cox regression analyses, we investigated whether the prognostic values of the six mRNA were independent of clinicopathological factors. Univariate Cox regression model showed that the risk score, race, TNM stage, N status, M status, tumour status, and radiation were significantly related to the patients’ overall survival in patients with TNBC (Table 4). In addition, multivariate Cox analysis indicated that the risk score and N stage still had remarkable independent prognostic values, with *P *= 0.005 and 0.025, respectively (Table 4). These results indicate that the 6-mRNA risk score was an independent prognostic indicator that can effectively predict the prognosis of TNBC patients.

**Table 4:** Univariate and multivariate Cox regression analysis of overall survival in TNBC

Variables	Univariate analysis			Multivariate analysis		
	HR	95%CI	*P*-value	HR	95%CI	*P*-value
Risk score (high vs low)	26.986	(3.487–208.858)	*0.002*	20.691	(2.551–167.789)	*0.005*
Age (≥ 54 vs < 54)	1.093	(0.442–2.700)	0.847			
TNM (I + II, III + IV)	4.283	(1.725–10.633)	*0.002*	1.131	(0.269–4.757)	0.866
T (T0 + 1, T2 + 3)	1.994	(0.711–5.592)	0.189			
N (N0 + 1, N2 + 3)	10.753	(3.236–35.733)	*<* *0.0001*	7.736	(1.287–46.486)	*0.025*
M (M0, M1)	37.815	(3.428–417.110)	*0.003*	8.963	(0.662–121.313)	0.099
Tumour status (no vs yes)	42.311	(5.453–328.319)	*<* *0.0001*			
Race (non-white vs white)	0.354	(0.140–0.896)	*0.028*	0.404	(0.129–1.270)	0.121
Radiation (no vs yes)	6.480	(1.751–23.979)	*0.005*			
Targeted (no vs yes)	2.494	(0.534–11.641)	0.245			

Italic values indicate significance of *P* value (*p* < 0.05)

HR: Hazard ratio; CI: confidence interval

## Discussion

TNBC is characterized as a complex and aggressive disease with poor survival rates compared with other subtypes. Only 30% to 45% of TNBC patients achieve a complete pathological response and survival rates similar to other breast cancer subtypes [21]. The poor prognosis of patients diagnosed with TNBC is mainly due to a lack of effective targets for treatment. Therefore, there is an urgent need for more effective therapeutic targets to improve TNBC prognosis.

Misregulation of the cell cycle is a hallmark of cancer [22], disorders in mechanisms of cell cycle monitoring and proliferation cause tumour cell growth and tumour cell-specific phenomena. However, it remains unclear if misregulation of periodic mRNAs bears significance in TNBC patient pathogenesis. In this study, a total of 755 DEGs involved in TNBC were screened out from TCGA database, including 590 up-regulated and 165 down-regulated genes. We then built related PPI networks of these DEGs and identified a significant module related to cell cycle, including several key DEGs in the regulatory network of TNBC patients. Subsequently, we identified eight periodic core genes (CCNA2, CCNB2, CDC20, BUB1, TTK, CENPF, CENPA, and CENPE) in the PPI network with higher capacity for PPIs. Coincidentally, all of them were up-regulated genes in TNBC (Fig. 5). CCNA2 (CyclinA2) and CCNB2 (CyclinB2) are members of the cyclin family of proteins that play key roles in the progression of G2/M transition, and have been reported to be the risk factors for resistance and recurrence [23–25]. Importantly, CCNA2, CCNB2, CDC20, BUB1, TTK, CENPA, and CENPE have been reported to be potential therapeutic targets for TNBC [26–29], and TTK inhibitors are currently being evaluated as anticancer therapeutics in clinical trials. These trends are highly consistent with our findings. However, there is no relevant report on CENPF in relation to TNBC; CENPF may be related in patient pathogenesis and as a novel potential therapeutic TNBC target.

Clinical pathological features (Additional file 2: Table S4) are the proper prognostic references for TNBC patients. However, recent studies have demonstrated that clinical predictors are insufficient to precisely predict patient disease outcomes. The mRNA prognostic biomarker has the robust capacity of predicting the survival status of cancer patients. For example, Papadakis et al. [30] confirmed that mRNA BAG-1 acts as a biomarker in early breast cancer prognosis, Zheng et al. [31] found that CBX2 is a potential prognostic biomarker and therapeutic target for breast cancer.

However, it is insufficient as the single gene marker to independently predict patient survival. Because a single gene is easily affected by various factors, it is difficult to provide a stable and effective prediction effect. Therefore, we used Cox model analysis to construct a gene signature that includes several genes to enhance prognostic prediction efficiency and sensitivity to TNBC. It has been widely confirmed that combined genetic models are superior to previous single gene markers in disease prediction and diagnoses [32].

In this study, we constructed a six-mRNA (TMEM252, PRB2, SMCO1, IVL, SMR3B and COL9A3) signature for efficient and sensitive prognosis of TNBC patients. A previous study reported that COL9A3 potentially contributes to the pathogenesis of canine mammary tumours [33]. In another study, using RNA-seq to identify diabetic nephropathy, the expression of TMEM252, increased in diabetic patients relative to wild-type controls [34], but we have not found any relevant studies of TMEM252 in tumours. PRB2 is a key factor in regulating ER gene expression. In MCF-7 cells, PRB2 can interact with ER-beta to interfere with ER-beta shuttle between nuclear and cytoplasm [35], whereas ER-α gene inactivation is mediated by PRB2 in ER-negative breast cancer cells [36]. These findings suggest that PRB2 may be considered a promising target for TNBC therapy. Only one NCBI article was found to study the function of the single-pass membrane protein with coiled-coil domains 1 (SMCO1), which may contribute to hepatocyte proliferation and have the potential to promote liver repair and regeneration [37]. However, we have not found any research on SMCO1 in breast cancer; we speculate that it may also play an important role in breast cell proliferation. Additionally, we are not aware of any specific study on SMR3B in tumours, but SMR3B amplification has been detected in osteopontin (OPN)-positive hepatocellular carcinoma [38]. Involucrin (IVL), a component of keratinocyte crosslinked envelope, is found in the cytoplasm and crosslinked with membrane proteins by transglutaminase. This gene is mapped to 1q21, among calpactin I light chain, trichohyalin, profillaggrin, loricrin, and calcyclin. However, to our knowledge, there is no research on IVL in TNBC.

As far as we know, this is the first established 6-mRNA signature for the prediction of OS time in TNBC, and we have demonstrated the independent prognostic value of this 6-mRNA signature in TNBC.

## Conclusions

In summary, through bioinformatic analysis, we identified eight hub genes, correlated with cell cycle, that might be tightly correlated with TNBC pathogenesis. Besides, we constructed a 6-mRNA signature which may act as a potential prognostic biomarker in patients with TNBC, and the prognostic model presented a good performance in OS prediction at 3 and 5 years. These findings will provide some guidance for future TNBC prognosis and molecular targeted therapy. However, our research is based on data analysis, and biological experiments are urgently needed to verify the biological roles of these predictive mRNAs in TNBC.

## Additional files

**Additional file 1: Table S1.** Differential expressed mRNAs between 1109 breast cancer tissue samples and 113 normal tissue samples. Differential expressed mRNAs between 117 TNBC and 970 non-TNBC breast cancer samples. 590 up-regulated overlapped DEGs and 165 down-regulated overlapped DEGs.

**Additional file 2: Table S2.** Functional and pathway enrichment analysis of DEGs in TNBC. **Table S3.** Prognostic value of the 16 genes by univariate Cox regression. **Table S4.** Clinical pathological parameters of patients with TNBC.

**Additional file 3: Fig. S1.** Validation of the 8 hub genes correlated with cell cycle in GEO dataset. Expression values of genes are log2-transformed.

## Data Availability

The datasets generated and/or analysed during the current study are available in The Cancer Genome Atlas database and additional files.

## Abbreviations

TNBC: triple-negative breast cancer
GEO: gene expression omnibus
ER: estrogen receptor
PR: progesterone receptor
HER2: human epidermal growth factor receptor 2
DEG: differentially expressed gene
TCGA: The Cancer Genome Atlas
PPI: protein–protein interaction
RFS: relapse-free survival
MF: molecular functions
BP: biological processes
CC: cellular components
