# Supplementary material for: Identification of potential key genes and pathways predicting pathogenesis and prognosis for triple-negative breast cancer
Source: Cancer Cell Int. 2019 Jun 28;19:172. doi: 10.1186/s12935-019-0884-0 (PMC6599314; doi:10.1186/s12935-019-0884-0)
Supplement: Supplementary file 2 — Additional file 2: Table S2. Functional and pathway enrichment analysis of DEGs in TNBC. Table S3. Prognostic value of the 16 genes by univariate Cox regression. Table S4. Clinical pathological parameters of patients with TNBC. [file 12935_2019_884_MOESM2_ESM.docx]

**Table S2** **Functional and pathway enrichment analysis of DEGs in TNBC**

| **Category** | **Term** | **Description** | **Count** | ***P*-value** |
| --- | --- | --- | --- | --- |
| BP term | GO:0007067 | mitotic nuclear division | 25 | 1.55E-8 |
| BP term | GO:0007062 | sister chromatid cohesion | 16 | 3.88E-8 |
| BP term | GO:0051301 | cell division | 26 | 2.60E-6 |
| BP term | GO:0060048 | cardiac muscle contraction | 9 | 1.34E-5 |
| BP term | GO:0030049 | muscle filament sliding | 8 | 3.70E-5 |
| BP term | GO:0051310 | metaphase plate congression | 5 | 1.61E-4 |
| BP term | GO:0001501 | skeletal system development | 13 | 1.66E-4 |
| BP term | GO:0007059 | chromosome segregation | 9 | 2.80E-4 |
| BP term | GO:0007080 | mitotic metaphase plate congression | 7 | 2.80E-4 |
| BP term | GO:0006366 | transcription from RNA polymerase II promoter | 27 | 5.23E-4 |
| CC term | GO:0005576 | extracellular region | 74 | 4.22E-7 |
| CC term | GO:0000775 | chromosome, centromeric region | 11 | 1.31E-6 |
| CC term | GO:0000776 | kinetochore | 12 | 5.34E-6 |
| CC term | GO:0005615 | extracellular space | 59 | 3.40E-5 |
| CC term | GO:0030017 | sarcomere | 8 | 4.50E-5 |
| CC term | GO:0005578 | proteinaceous extracellular matrix | 20 | 4.82E-5 |
| CC term | GO:0031672 | A band | 5 | 3.17E-4 |
| CC term | GO:0005871 | kinesin complex | 8 | 3.34E-4 |
| CC term | GO:0005859 | muscle myosin complex | 5 | 5.54E-4 |
| CC term | GO:0030016 | myofibril | 6 | 5.85E-4 |
| MF term | GO:0003777 | microtubule motor activity | 9 | 9.12E-4 |
| MF term | GO:0001077 | transcriptional activator activity | 16 | 0.001 |
| MF term | GO:0043565 | sequence-specific DNA binding | 26 | 0.001 |
| MF term | GO:0005198 | structural molecule activity | 16 | 0.001 |
| MF term | GO:0008009 | chemokine activity | 6 | 0.001 |
| MF term | GO:0008017 | microtubule binding | 14 | 0.002 |
| MF term | GO:0003682 | chromatin binding | 16 | 0.002 |
| MF term | GO:0003779 | actin binding | 14 | 0.024 |
| MF term | GO:0008307 | structural constituent of muscle | 6 | 0.003 |
| MF term | GO:0003774 | motor activity | 7 | 0.006 |
| KEGG  pathway | hsa04110 | Cell cycle | 14 | 2.56E-6 |
| KEGG  pathway | hsa03320 | PPAR signaling pathway | 8 | 5.92E-4 |
| KEGG  pathway | hsa04970 | Salivary secretion | 8 | 0.002 |
| KEGG  pathway | hsa04974 | Protein digestion and absorption | 8 | 0.002 |
| KEGG  pathway | hsa04727 | GABAergic synapse | 7 | 0.010 |
| KEGG  pathway | hsa04152 | AMPK signaling pathway | 8 | 0.017 |
| KEGG  pathway | hsa04920 | Adipocytokine signaling pathway | 6 | 0.017 |
| KEGG  pathway | hsa00980 | Metabolism of xenobiotics by cytochrome P450 | 6 | 0.022 |
| KEGG  pathway | hsa04260 | Cardiac muscle contraction | 6 | 0.023 |
| KEGG  pathway | hsa04114 | Oocyte meiosis | 7 | 0.033 |

Note: Top 10 terms were selected according to P-value.

Abbreviations:BP, biological process; CC, cellular component; DEG, differentially expressed gene; GO, Gene Ontology; KEGG, Kyoto Encyclopedia of Genes and Genomes; MF, molecular function.

**Table S3 Prognostic value of the 16 genes by the univariate Cox regression**

| **gene** | **HR** | **z** | ***P* value** |
| --- | --- | --- | --- |
| ZNF971P | 14.68739 | 3.081587 | 0.002059 |
| SMR3B | 1.125379 | 2.648327 | 0.008089 |
| INA | 0.772502 | -2.46971 | 0.013522 |
| COL9A3 | 0.789441 | -2.36945 | 0.017815 |
| IGHGP | 0.802313 | -2.32249 | 0.020207 |
| CACNA1S | 1.588408 | 2.30459 | 0.02119 |
| DLX6 | 0.803978 | -2.27519 | 0.022895 |
| SMCO1 | 1.848173 | 2.242285 | 0.024943 |
| OFCC1 | 0.745327 | -2.24041 | 0.025064 |
| TMEM252 | 1.452054 | 2.233507 | 0.025516 |
| PRB4 | 1.298 | 2.220213 | 0.026404 |
| PRB2 | 1.263275 | 2.146307 | 0.031849 |
| TCAM1P | 0.851121 | -2.10642 | 0.035168 |
| IGHG1 | 0.842163 | -2.09655 | 0.036034 |
| CLDN6 | 0.790798 | -2.07538 | 0.037952 |
| COL9A1 | 0.855457 | -2.07492 | 0.037994 |
| IVL | 1.135495 | 2.066787 | 0.038754 |

| **characteristic** | **n** | **Risk score level** | | |
| --- | --- | --- | --- | --- |
|  |  | **Low** | **High** | ***P* value** |
| Age(years) |  |  |  | 0.407 |
| ≥54 | 61 | 33 | 28 |  |
| <54 | 56 | 26 | 30 |  |
| TNM stage |  |  |  | 0.058 |
| I and II | 92 | 50 | 42 |  |
| III and IV | 22 | 7 | 15 |  |
| T |  |  |  | 0.569 |
| T0+1 | 102 | 52 | 50 |  |
| T2+3 | 14 | 6 | 8 |  |
| N |  |  |  | **0.029** |
| N0+1 | 101 | 55 | 46 |  |
| N2+3 | 16 | 4 | 12 |  |
| M |  |  |  | 0.311 |
| M0 | 110 | 56 | 54 |  |
| M1 | 1 | 0 | 1 |  |
| Tumor status |  |  |  | **<0.0001** |
| Tumor free | 85 | 51 | 34 |  |
| With tumor | 15 | 1 | 14 |  |
| Race |  |  |  | 0.121 |
| Non-white | 29 | 11 | 18 |  |
| White | 88 | 48 | 40 |  |
| Radiation |  |  |  | 0.398 |
| Yes | 60 | 33 | 27 |  |
| No | 45 | 24 | 21 |  |
| Targeted |  |  |  | 0.277 |
| Yes | 80 | 40 | 40 |  |
| No | 7 | 5 | 2 |  |

**Table S4 Clinical pathological parameters of patients with TNBC**
