# Supplementary figures and images for: Identification of potential key genes and pathways predicting pathogenesis and prognosis for triple-negative breast cancer
Source: Cancer Cell Int. 2019 Jun 28;19:172. doi: 10.1186/s12935-019-0884-0 (PMC6599314; doi:10.1186/s12935-019-0884-0)

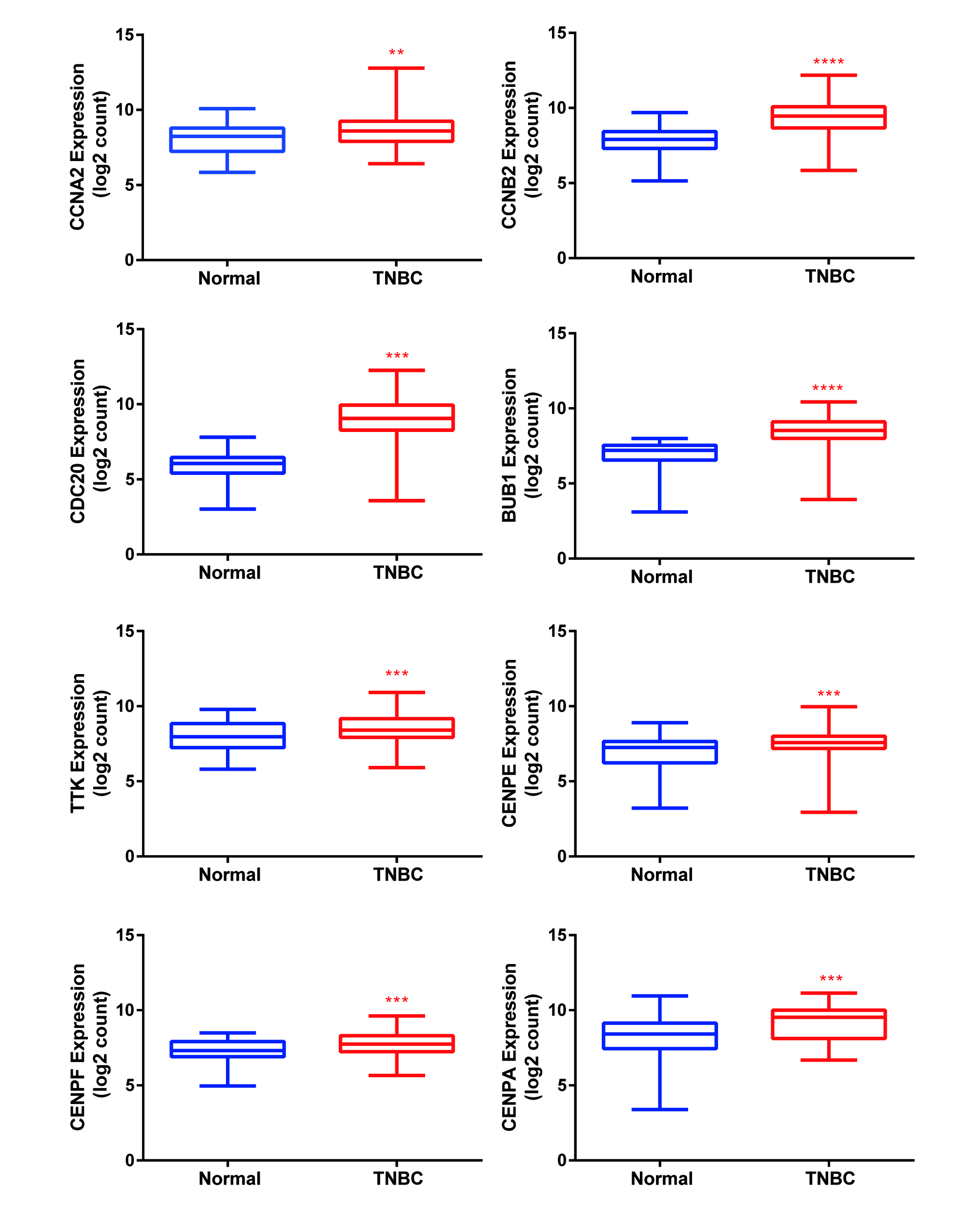

Supplement: Supplementary file 3 — Additional file 3: Fig. S1. Validation of the 8 hub genes correlated with cell cycle in GEO dataset. Expression values of genes are log2-transformed. [file 12935_2019_884_MOESM3_ESM.tif]
